# Anterior Subluxation of a Metal-on-Metal Total Hip Arthroplasty Resulting in Erosion and Metal Debris

**DOI:** 10.1155/cro/3718607

**Published:** 2025-02-20

**Authors:** Connor Park, Jens Verhey, Roman Austin, Daniel Howgate, Abhijith Bathini, Mark K. Lyons, Joshua S. Bingham

**Affiliations:** ^1^Creighton University School of Medicine-Phoenix, Phoenix, Arizona, USA; ^2^Department of Orthopedic Surgery, Mayo Clinic, Phoenix, Arizona, USA; ^3^Department of Neurosurgery, Mayo Clinic, Phoenix, Arizona, USA

## Abstract

**Introduction:** Total hip arthroplasty (THA) is a commonly performed and highly successful surgical procedure. Metal-on-metal (MoM) THA implants were introduced two decades ago and subsequently recalled due to high early revision rates. Acetabular cup erosion and fragmentation secondary to chronic edge loading causing delayed instability are rare but devastating complications of MoM THA warranting expeditious revision surgery.

**Case Presentation:** We report a 70-year-old male with a history of bilateral MoM THA who presented with left hip instability. In addition to the radiographic and clinical features of hip instability, macroscopic examination at revision surgery revealed extensive erosion and fragmentation of the antero-superior margin of the implanted cup, osteolysis, and widespread metallosis of the periarticular soft tissues.

**Discussion:** This case highlights a significant adverse complication of MoM THA. Despite the industry's wide discontinuation and recall of these implants, MoM hip arthroplasty implants are present in many patients, who are all at risk of developing similar complications. Guidelines for the surveillance and treatment of both symptomatic and asymptomatic MoM THAs have been reported, although ambiguity remains in the optimal approach for managing patients with existing MoM THA.

**Conclusion:** Failure of MoM hip arthroplasty is most commonly the result of adverse reaction to metal debris. We present a novel mechanism of failure in a patient presenting with late instability due to asymmetric wear of the MoM bearing surface.. While it is uncertain whether early intervention in this patient may have prevented this complication, arthroplasty surgeons should be aware of the various modes of failure for MoM hip implants, as expeditious revision surgery is often required.

## 1. Introduction

Total hip arthroplasty (THA) is a well-established, commonly performed and highly effective operation for end-stage degenerative joint disease [1]. Future demand for THA is projected to rise exponentially, with a fivefold increase predicted in patients under 55 by 2030 [2]. Despite the high levels of patient satisfaction and survivorship observed with contemporary THA designs and surgical techniques, revision surgery may occasionally be indicated. Recent literature reports that 21.3% of primary THAs are revised within 15 years, with 22.3% of secondary revisions undergoing a further revision within 7 years, and 19.3% of tertiary revisions are revised again within 3 years [3]. Common indications for undergoing revision surgery following primary THA include aseptic loosening, instability, periprosthetic joint infection (PJI), periprosthetic fracture (PPF), or adverse soft tissue reaction to particulate debris. The incidence of these conditions is themselves influenced by a complex interaction of both the patient and surgical factors. However, some of the most important surgical factors to consider include the interval from index operation, bearing surface and head size used, and implant fixation. The use of uncemented or hybrid implant fixation combined with either ceramic or metal on highly cross-linked polyethylene bearings is the most prevalent in modern THA practices, although notable variations exist in surgical, institutional, and geographical practices worldwide [4].

Although polyethylene bearing surface implants have predictable and adequate survivability, degradation of the polyethylene liner may lead to osteolysis and aseptic loosening over time, especially in prior generation polyethylene manufacturing [5]. Similarly, the advantages of ceramic materials, including their notably low friction and wear rates, are counterbalanced by their fracture potential [4]. Many of the issues with these combinations were thought to be potentially remedied by metal-on-metal (MoM) implants.

The modern MoM gained popularity in the early 2000s because of its theoretical low wear rate. Additionally, the theoretical low wear rate of the MoM implant allowed for the utilization of larger head sizes, conferring additional stability when compared to traditional metal on polyethylene bearing surface [6]. These implants were subsequently recalled from the market in 2010 due to their high five-year rate of revision history [7]. Additionally, the production of particulate metal ions as a result of surface wear from these implants has been found to result in high serum concentrations of metal ions and adverse local tissue reaction [8]. Elevated levels of cobalt from a bilateral MoM THA have been associated with serious systemic toxicity such as progressive cardiomyopathy [9]. The currently recommended approach for revision involves utilizing a center of pressure (CoP) implant with the largest feasible head size to minimize postoperative dislocation [8, 10, 11].

## 2. Case Presentation

### 2.1. Patient History

The patient is a 72-year-old male with a history of hypertension, gastroesophageal reflux disease (GERD), coronary artery disease (CAD) status post coronary artery bypass grafting, myocardial infarction, peripheral arterial disease, and paroxysmal atrial fibrillation who initially presented with left hip pain and difficulty ambulating for 2 weeks. The patient underwent staged, bilateral THAs via a posterior approach. His recoveries from index procedures were uneventful, as he denied any history of perioperative medical or surgical complications, including wound dehiscence, prior instability events, or pain. The patient had several years of appropriate function and pain-free mobility.

He was initially evaluated at an outside clinic for 2 weeks of insidious-onset left hip pain. Radiographs demonstrated an anterior hip dislocation, and he was instructed to visit the emergency department. He denied any trauma or falls prior to onset of pain. Furthermore, he denied any pain or mechanical symptoms affecting the contralateral hip. Physical examination showed shortening of the left lower extremity (LLE). An attempt was made to reduce the dislocation via closed reduction in the operating room under anesthesia which failed due to persistent subluxation.

### 2.2. Preoperative Investigations

**Figure 1:**
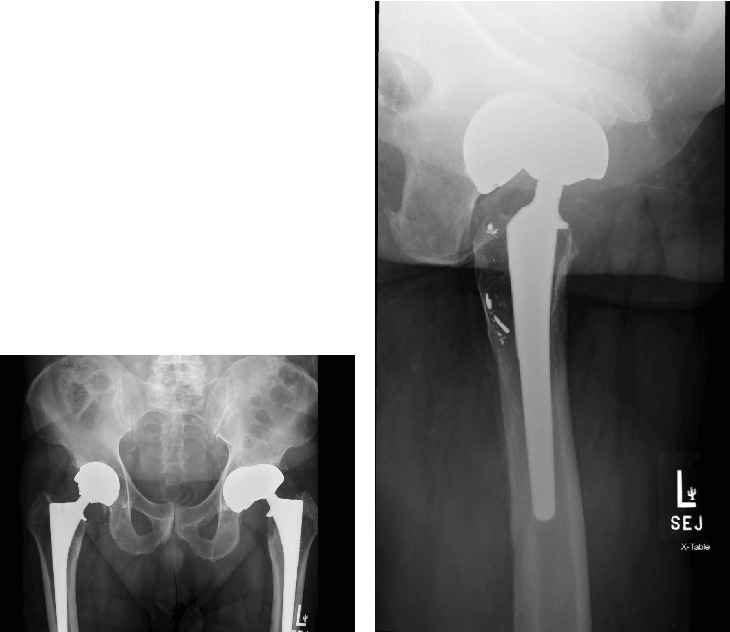
(a) Anterior–posterior (AP) radiograph demonstrating subluxation of the left THA. (b) Lateral radiograph of the left hip demonstrating anterior subluxation with metallic acetabular cup fragments situated in the area of osteolysis within the posterior aspect of the proximal femur.

**Figure 2:**
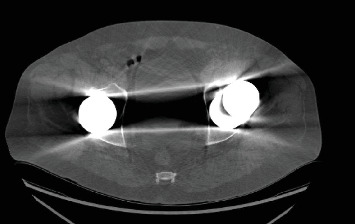
CT pelvis without contrast demonstrating increased acetabular component anteversion of the unstable left total hip arthroplasty.

The failed implant raised concerns about potential adverse reaction to metal. Therefore, serum metal ions, erythrocyte sedimentation rate (ESR), and C-reactive protein (CRP) laboratory tests were obtained demonstrating elevated cobalt and chromium levels of 299 and 114, respectively. ESR and CRP were within normal limits. An aspiration of the left hip was also performed, but cell count was unable to be obtained due to cell degeneration and necrosis. Alpha defensin from synovial aspirate was negative. Follow-up radiographs showed extensive erosion of the anterior-superior portion of the cup resulting from continual anterior subluxation. MRI was ordered to evaluate the soft tissue envelope and assess for a pseudotumor, while CT was also obtained to assess for bony defects in preparation for revision. MRI pelvis demonstrated small joint effusion without evidence of a pseudotumor. CT without contrast of the affected hip demonstrated proximal femoral osteolysis and subacute fracture of the anterior column. Fragments from the margin of the eroded cup were found to have migrated into the proximal femur (Figures 1 and 2).

### 2.3. Surgical Technique

**Figure 3:**
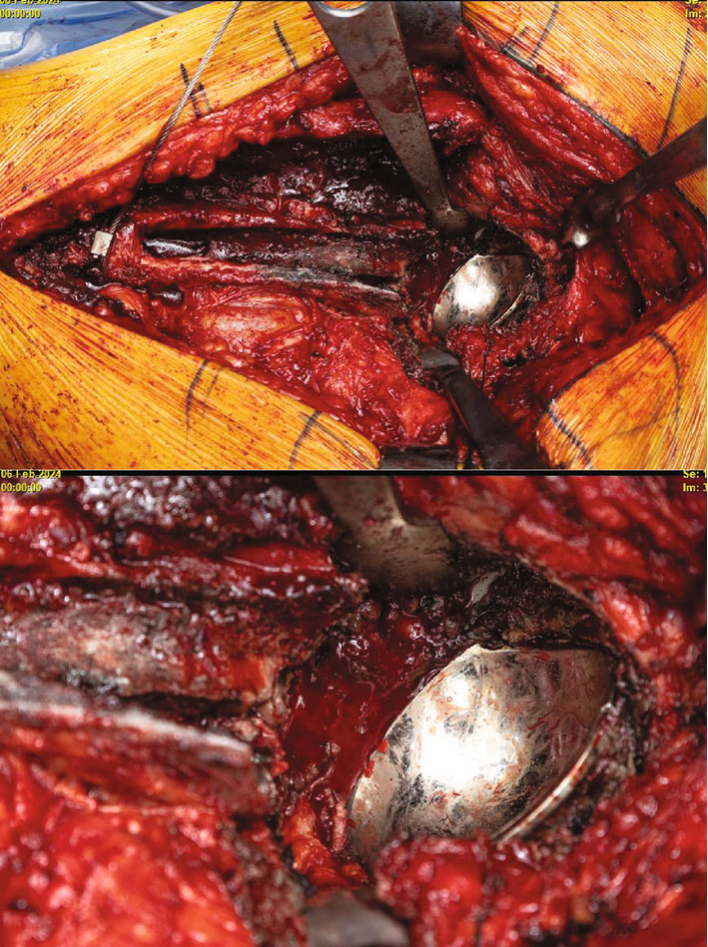
Metallosis within the periarticular soft tissues of the left hip alongside marginal erosion and fragmentation of the acetabular cup.

At the time of revision, a posterolateral approach was used, and the prior incision was excised. Upon dissection, there was a notable amount of metallosis around the left hip joint that was thoroughly debrided and removed (Figure 3). Additionally, three samples from deep tissue were sent to be cultured. Cultures were negative, but the gram stain returned positive for gram-negative bacillus. This was deemed to be false positive due to high levels of serum cobalt and chromium in addition to the staining process during the gram stain. The originally implanted head in the first THA was inseparable from the stem due to cold welding. The decision was then made to perform an extended trochanteric osteotomy to remove the well-fixed femoral stem.

**Figure 4:**
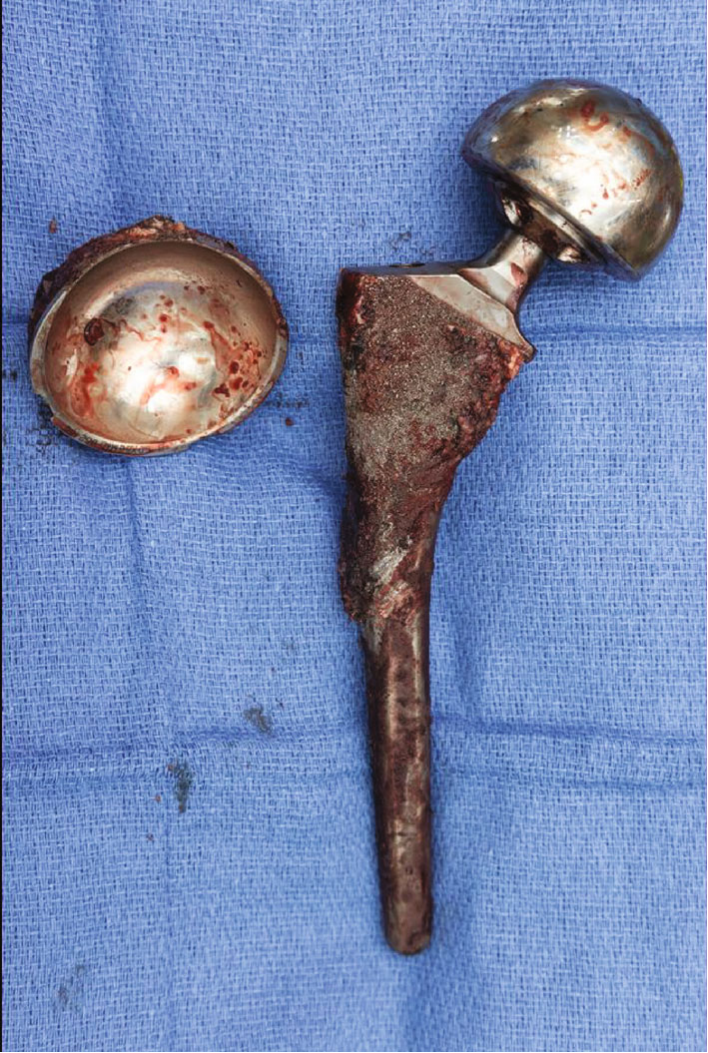
Explanted acetabular and femoral prostheses.

**Figure 5:**
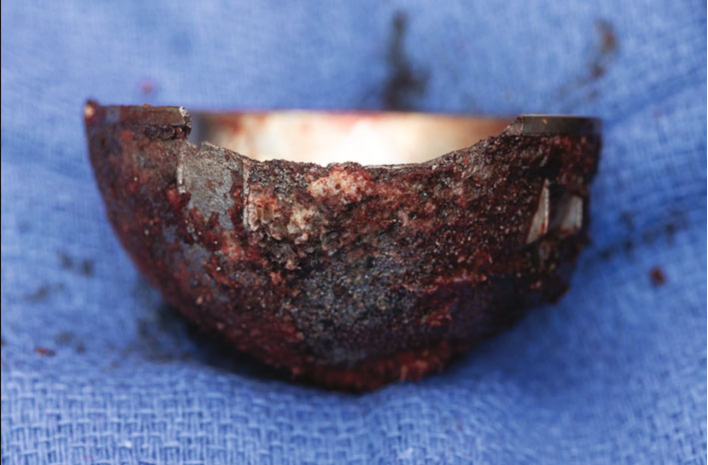
Explanted acetabular cup demonstrating extensive marginal erosion and fragmentation.

**Figure 6:**
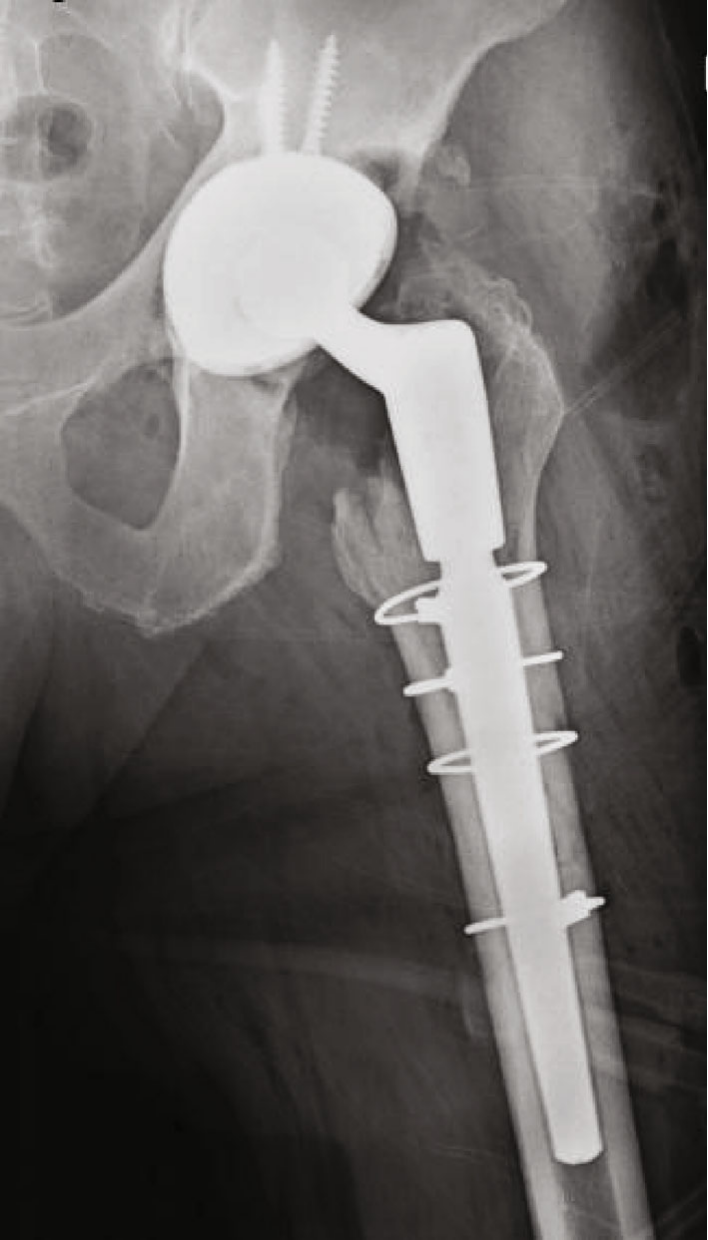
Intraoperative radiograph following successful reduction and component revision.

After removal, the anterior and superior erosion was visible in the margin of the cup (Figures 4 and 5). The area was subsequently reamed up to allow for the insertion of a new 62-mm Biomet G7 cup. This was followed by the placement of three screws and a dual-mobility liner. For the femur, a prophylactic cable was inserted before placement of the 20-mm distal body portion of a Stryker restoration modular stem. Following this, intraoperative radiographs were obtained to confirm accurate alignment and positioning. The hip was then dislocated, and the real proximal 25-mm body was inserted and torqued down. A 50-mm dual-mobility head was inserted, and the hip was reduced (Figure 6).

Hemostasis was obtained, and the wound was thoroughly irrigated with dilute betadine. The osteotomy was closed with three additional cerclage cables. The vastus was closed with running 0 suture. The posterior capsule was closed with five Ethibond through bone tunnels. The patient was placed in a hip abduction brace before being extubated.

### 2.4. Postoperative Course

The patient tolerated the procedure well. There was an expected hemoglobin drop postoperatively, and he did not require any transfusion. Given the requirement for extended trochanteric osteotomy for femoral component extraction, the patient was instructed to maintain toe touch weight bearing for 12 weeks. The patient was anticoagulated with Lovenox throughout his hospital stay. His IV fluids and narcotic medications were weaned down within the first 24 h. The following day, the patient was afebrile, his pain was controlled with oral medication, and the remainder of his vital signs were normal; therefore, he was deemed satisfactory for discharge home with home healthcare on post-operative Day 1.

### 2.5. Aftercare and Follow-Up

Written consent was obtained from the patient to publish medical history, treatment course, and relevant clinical imaging and radiographs in accordance with the institutional protocol. All clinical information is deidentified. The patient was seen in the clinic at 1 week and 2 weeks following surgery and reported that he was doing well overall. His incision was intact, and negative pressure wound therapy was discontinued. The patient was advised to continue toe touch weight bearing and wearing his hip abduction brace and further counseled on avoiding positions causing risk of dislocation. His pain was well controlled at this point.

The patient presented at his 6- and 12-week follow-up appointments stating that his function was gradually improving. His exam was overall unchanged from his prior visit, and his wound was continuing to heal appropriately without evidence of infection. Follow-up imaging was obtained demonstrating satisfactory component positioning. Additionally, the extended trochanteric osteotomy site was healing in satisfactory alignment. The patient was advised to progress with weight bearing as tolerated with the use of a walker. His brace was discontinued. His heavy metal ions will be serially monitored every 6 months, and the patient was given specific instructions to return should his contralateral THA become symptomatic.

## 3. Discussion

Patients with MoM can be separated into two groups to guide management: asymptomatic and symptomatic. Follow-up and treatment of asymptomatic patients remain controversial. It is advisable to measure metal ion levels in asymptomatic patients with risk factors such as malpositioned components and radiographic changes suggestive of osteolysis or loosening and those with recalled implants [8]. Annual follow-up with plain radiographs is appropriate for asymptomatic patients without risk factors [8]. Various organizations, including the UK MHRA, Hip Society, and Health Canada, have historically set a serum cobalt or chromium threshold of 7 ppb to guide management decisions. However, some experts suggest that a lower cutoff, such as 5 ppb, might be more appropriate [8].

Annual follow-up with serum metal ion tests and plain radiographs is suitable for patients with low metal ion levels, whereas advanced imaging is recommended for those with elevated levels [8]. There does not appear to be a consensus regarding optimal advanced imaging around an implant. Advanced imaging modalities such as ultrasound or metal artifact reduction sequence (MARS) MRI are generally recommended to assess soft tissue lesions [8, 11]. For patients with elevated ion levels and no soft tissue lesions, close follow-up in 3–6-month intervals is advisable [8]. The management of asymptomatic patients with early signs of adverse reaction to metal debris (ARMD) is particularly challenging. In such cases, thorough consultations are essential to assess the need for revision surgery, particularly when there are increasing ion levels, progressive radiographic changes, or emerging symptoms [8]. Patients without a history of revision surgical intervention are recommended to be followed every 3–6 months [8].

Surgeons should carefully evaluate symptomatic patients with a thorough history and physical exam to rule out other causes of hip pain. Radiographs should be taken to evaluate for component position, signs of edge loading or impingement, signs of osteolysis, or implant loosening [11]. Previously, high cup inclination and large femoral head size have been shown to increase metal ion production secondary to edge loading and volumetric wear. Authors have suggested that attention should be directed to the retroacetabular, ischial, and pubic regions on patient radiographs [11]. Blood tests may reveal elevated ESR or CRP, which, while commonly associated with ARMD, require cautious interpretation as they do not always indicate infection [11]. Synovial fluid analysis, particularly with manual cell counting, is often necessary, as automated counts can be falsely elevated by debris [11]. Advanced imaging with ultrasound or MRI is recommended in these individuals to further evaluate soft tissue structures [11]. Symptomatic individuals with low metal ion levels and no evidence of abnormal results upon advanced imaging are recommended to be monitored every 3–6 months with additional radiographs and serum metal ion levels [8]. Symptomatic patients with elevated serum cobalt and chromium levels in combination with abnormal advanced imaging results constitute the highest risk group and should be considered for immediate revision surgery [8].

The high failure rates of MoM implants have been largely associated with metallosis, formation of pseudotumors, and adverse tissue reaction to metal debris [6]. Although the high 5-year revision rate led to a discontinuation of MoM implants in 2010, patients continue to present for revision today. Despite this, retrospective research on the MoM Biomet M2a-38 implant showed that these patients have done surprisingly well, with 20-year implant survivorship of 97.6% and very few instances of failures [12].

Currently, it is widely agreed upon that implant failure resulting in dislocation or pseudotumor formation warrants immediate revision. There is a concern for systemic toxic metal ion levels due to metallosis from failed implants. It is imperative to also recognize the shortened viability of each subsequent revision in a patient. Initially, the prevailing belief was that revisions due to ARMD had a higher rate of another subsequent revision. Consequently, surgeons lowered the threshold of criteria for an initial revision of an MoM THA due to ARMD. The rationale was to preemptively mitigate later stage degradation and reaction to debris which would cause the need for another revision. Retrospective data has since refuted this belief and demonstrated that the highest risk of needing a subsequent revision was due to infection or dislocation/subluxation of the initial MoM [13].

As time progresses from the recall, one of the pertinent avenues of research needed to be explored is the danger involved in elevated blood metal ion concentrations. A large contingency of patients with MoMs who are asymptomatic with moderately elevated levels have not undergone revision. This is likely due to the rare occurrence of systemic disease from elevated metal ion levels, although cardiac failure secondary to cobalt toxicity from MoM has been reported [13]. Despite the rarity of presentation with systemic toxicity from metal ions, asymptomatic individuals have been found to have histologically significant tissue destruction around an MoM [14]. In this case, our patient had elevated levels of metal ion concentration and presented acutely with symptoms of toxicity. The lack of clarity regarding how patients, such as our own, may symptomatically be affected over time emphasizes the importance of extended follow-up.

Our presented case was one that anatomically warranted revision. The erosion seen in this patient through their acetabular cup is an infrequent complication. Penetration of the acetabular cup is usually seen in a superior–posterior manner [15]. This correlates with the majority of postoperative THA dislocation from a posterior approach occurring in the posterior direction [16]. However, in our presented case, there was penetration through the circumferential margin of the acetabular cup in an anterior and superior direction despite an initial posterior approach for the original implant. This resulted in additional erosion into the ilium located superior to the cup. Although initial radiographic implant positioning was unavailable from the index procedure, excessive anteversion of the acetabular cup is a recognized factor contributing to anterior dislocation. Radiographic imaging prior to revision was suggestive of excessive anteversion of the acetabular component as a cause of the observed atypical erosion. Furthermore, the absence of anterior erosion in the contralateral implant supports malpositioning as the cause, rather than a typical complication of MoM THA.

Revision surgery for MoM THA is a complex and individualized process, dependent on the condition of the implants and surrounding tissues [11]. Patients with well-fixed and well-positioned implants may require only limited revision involving exchange of modular components. In contrast, cases with malpositioned implants or significant soft tissue defects necessitate more extensive reconstruction. Surgeons must remain vigilant for infection, as MoM prostheses have been associated with increased infection rates possibly due to the presence of necrotic tissue and metal debris hindering immune system function, which can accelerate bacterial growth and antibiotic resistance [11].

Revisions of MoM THA pose substantial risks, including complications like dislocation, inadequate ingrowth of new components, bone loss, increased morbidity, blood loss, and longer operative times [8]. Re-revisions are often needed due to infection, dislocation, or failure of component ingrowth [8]. Abductor deficiency is common in these patients as a consequence of the cytotoxic effect of the metal ions [8]. Surgeons may address abductor deficiency with constrained liners, dual-mobility bearings, or even muscle transfers in severe cases [8]. To mitigate complications, alternative materials like ceramic on polyethylene articulations or ultraporous cups are being explored to reduce infection rates and aseptic loosening [8].

## 4. Conclusion

The attempted implementation of MoM hip replacements represents an interesting shortcoming in the field of orthopedics. Not only was revision required at a higher rate than comparative materials, but there is continued uncertainty on how to manage existing implants. Our case presents an uncommon subluxation leading to an anterior erosion pattern and metal debris. This patient falls under the category of individuals who are most at risk for additional revision and failure of this current revision [13]. It is important to consider whether earlier identification of metal debris or any adverse reaction could have prevented degradation of bony architecture. Continued retrospective analysis will be key in definitively understanding when a patient with an MoM qualifies for revision and the optimal approach to care.

## Data Availability

The data supporting this report are available from the corresponding author (M.K.L.) upon any reasonable request.
